# Response to: Comment on “Pretreatment Hepatitis C Virus NS5A/NS5B Resistance-Associated Substitutions in Genotype 1 Uruguayan Infected Patients”

**DOI:** 10.1155/2019/2520302

**Published:** 2019-02-05

**Authors:** Fabián Aldunate, Natalia Echeverría, Daniela Chiodi, Pablo López, Adriana Sánchez-Cicerón, Alvaro Fajardo, Martín Soñora, Juan Cristina, Nelia Hernández, Pilar Moreno

**Affiliations:** ^1^Laboratorio de Virología Molecular, Centro de Investigaciones Nucleares, Facultad de Ciencias, Universidad de la República, 11400 Montevideo, Uruguay; ^2^Clínica de Gastroenterología, Hospital de Clínicas, Facultad de Medicina, Universidad de la República, 11600 Montevideo, Uruguay

We are very grateful to Dr. Sana Eybpoosh and Dr. Mona Kiminezhad for their valuable comments and suggestions [1] on our recent article entitled “Pretreatment Hepatitis C Virus NS5A/NS5B Resistance-Associated Substitutions in Genotype 1 Uruguayan Infected Patients” [2].

We have carefully reviewed all the comments [1] and hope that the provided responses will address the concerns on validity and generalizability of our results.

The first concern raised by Eybpoosh and Kiminezhad refers to the possibility that our samples may have reached partial or full “substitution saturation” given the fact that they were collected from chronic hepatitis C virus (HCV) cases. As stated, this phenomenon of saturation occurs more rapidly in fast evolving pathogens [3] and it is well established that HCV exhibits a fast evolutionary rate ranging from 10^−3^ to 10^−4^ substitutions/site/year [4–6]. As substitution saturation could decrease the phylogenetic signal in the sequence alignment, the authors suggest that it could affect the quality and accuracy of our phylogenetic inferences. However, likelihood mapping studies had previously been performed on all of our datasets in order to determine the phylogenetic signal of our aligned sequences [7]. Unfortunately, we did not include these results in our publication. In all cases, the likelihood mapping results confirmed that over 91% of the trees rendered fully resolved topologies (Figure 1). Our NS5A and NS5B datasets for genotyping purposes (Figure 1 in [2]) included 31 Uruguayan strains and 58 or 59 strains, respectively, corresponding to different HCV subtypes and genotypes isolated elsewhere. In particular, as shown in Figure 1(a), our NS5A dataset (953 nucleotide long) showed that 93.8% of trees are fully resolved, whereas for our NS5B dataset (361 nucleotide long) this percentage was slightly lower (91.9%, Figure 1(b)), but in both cases, these results suggested sufficient phylogenetic signal for our subsequent analyses. The NS5A genotype 1a dataset, used to analyse evolutionary relationships (Figure 2 in [2]), included 20 Uruguayan HCV subtype 1a strains and 237 HCV subtype 1a strains isolated elsewhere (1119 nucleotide long). This dataset showed the highest percentage of fully resolved trees (95.5%, Figure 1(c)).

Despite the abovementioned results, we agree with the comment of the authors that we could have assessed the substitution saturation of our data. Therefore, we have addressed this issue using DAMBE [8], as suggested by them.  Figure 2 shows the results obtained for the Uruguayan strains when plotting the observed transitions and transversions of all codon positions against a GTR-corrected genetic distance (NS5A, Figure 2(a) and NS5B, Figure 2(b)). These results indicate that our sequences, derived from chronically infected patients, have not yet reached substitution saturation which is evidenced by a higher rate of transitions than transversions, both increasing with the genetic distance.

In addition to analysing Uruguayan strains, we have tested the substitution saturation in all of our datasets using Xia's method [9, 10]. This test provides an index of substitution saturation (I_ss_) and compares it with a critical I_ss_ (I_ss.c_) which corresponds to a value at which the sequences will begin to fail to recover the true tree. In addition, it is performed assuming two extreme tree topologies (symmetrical vs. asymmetrical). Since the computer simulation is limited to *n* ≤ 32 sequences and our datasets exceed this number, DAMBE randomly samples subsets of 4, 8, 16, and 32 sequences multiple times and performs the test for each subset. When considering NS5A genotype 1a dataset for evolutionary relationships (257 sequences), no saturation was found (Table 1). This is evidenced by I_ss_ values significantly smaller than I_ss.c_ for both tree topologies which argues for a dataset with little substitution saturation [10]. This, in turn, supports the evolutionary relationships inferred in Figure 2 in [2]. When performing Xia's method on NS5A and NS5B datasets for genotyping purposes (89 and 90 sequences, respectively, corresponding to all known HCV genotypes and several subtypes), the results are slightly different. As shown in Table 2, only when sample subsets of 32 sequences are considered and under an extremely asymmetric tree topology assumption, the results suggest that our datasets would be poor for phylogenetic studies (I_ss_ > I_ss.c_, *p* > 0.05). Nevertheless, it is worth noting that our likelihood mapping results (Figure 1) are indicative of good phylogenetic signal for both datasets. In addition, the topologies of the reconstructed phylogenetic trees (Figure 1 in [2]) seem to be more symmetric-like, and under the symmetric tree topology assumption, all I_ss_ values are significantly smaller than I_ss.c_, regardless of the number of sequences sampled. In conclusion, considering the tree topology as well as the likelihood mapping studies, the genotype assignment is presumably correct.

Eybpoosh and Kiminezhad also drew attention to the small number, apparently not randomly sampled, of HCV patients enrolled in our study. To better comprehend our sampling diversity, we must introduce some demographical data about our country. Uruguay comprises a small territory (176.215 m^2^) and has only about 3.3 million inhabitants [11]. To this respect, the Gastroenterology Clinic from Hospital de Clínicas, though located in Montevideo (the capital city), is the only university reference centre where patients from all over the country and belonging to different socioeconomic backgrounds are referred to. Hence, since the patients were recruited from this centre, we conceive our samples to be fairly representative of Uruguayan HCV-infected population. Nevertheless, we share the idea proposed by the authors [1] that having nationwide surveys would provide larger samples to support our studies. However, it is worth mentioning that such an approach would be very difficult to carry out in our country given that our HCV infection prevalence among general population is currently unknown [12]. In conclusion, following this line of thought, we believe that generalizations about the frequency of HCV resistance-associated substitutions (RASs) in the whole country are reasonably accurate. The purpose of our study was to contribute with an initial assessment of the presence of RASs to NS5A/NS5B inhibitors in a direct-acting antiviral agent treatment naïve cohort of Uruguayan patients chronically infected with HCV, and therefore, we encourage further studies on HCV resistance patterns.

Finally, we thank the authors for their comments and the editor for giving us the opportunity to clarify the concerns.

## Figures and Tables

**Figure 1:**
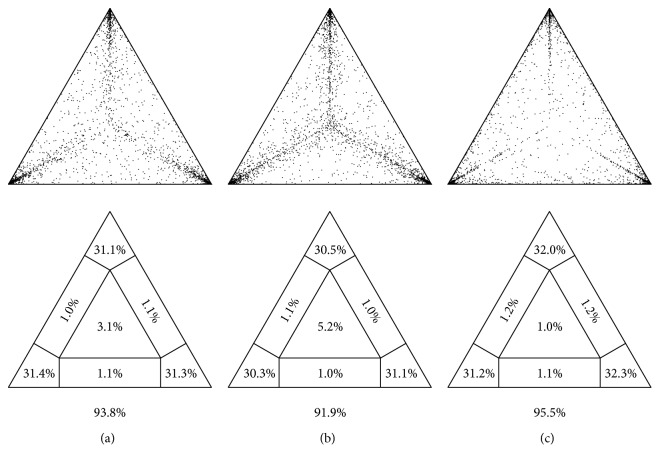
Likelihood mapping analysis. (a) NS5A dataset for genotyping purposes (*n* = 89 sequences, 953 nt); (b) NS5B dataset for genotyping purposes (*n* = 90 sequences, 361 nt); (c) NS5A genotype 1a dataset used to analyse evolutionary relationships (*n* = 257 sequences, 1119 nt). The analyses showed that, across all datasets, over 91% of trees are fully resolved (percentages are indicated below each triangle). Analyses were performed using 1000 random quartets and a GTR model of substitution with a mixed model of rate heterogeneity (1 invariable + 4 gamma rates).

**Figure 2:**
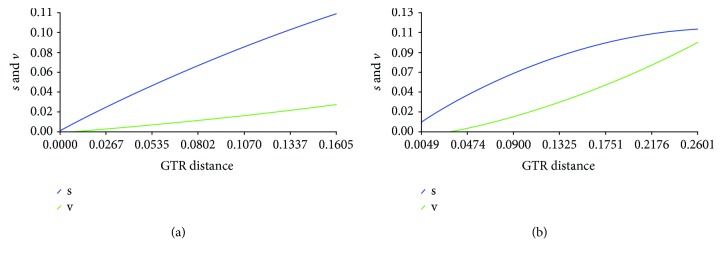
Substitution saturation analysis using DAMBE. (a) NS5A Uruguayan strain dataset; (b) NS5B Uruguayan strain dataset. Transitions (blue line) and transversions (green line) are plotted against divergence. The genetic distance was computed with the GTR nucleotide substitution model. The results show that all Uruguayan sequences included in our study [2] contain enough evolutionary information for reliable phylogenetic inferences and little substitution saturation despite corresponding to chronic HCV strains.

**Table 1:** Test of substitution saturation by Xia's method implemented in DAMBE [9, 10] on NS5A genotype 1a dataset used to analyse evolutionary relationships.

*n* sequences	I_ss_	Symmetrical topology		Asymmetrical topology	
		I_ss.c_Sym	*p*	I_ss.c_Asym	*p*
4	0.124	0.825	0.0000	0.793	0.0000
8	0.129	0.795	0.0000	0.691	0.0000
16	0.137	0.778	0.0000	0.585	0.0000
32	0.144	0.757	0.0000	0.458	0.0000

I_ss_: index of substitution saturation; I_ss.c_: critical index of substitution saturation; *p*: *p* value for two-tailed tests.

**Table 2:** Test of substitution saturation by Xia's method implemented in DAMBE [9, 10] on NS5A and NS5B datasets used for genotyping purposes.

	*n* sequences	I_ss_	Symmetrical topology		Asymmetrical topology	
			I_ss.c_Sym	*p*	I_ss.c_Asym	*p*
NS5A	4	0.434	0.819	0.0000	0.787	0.0000
	8	0.427	0.787	0.0000	0.681	0.0000
	16	0.433	0.77	0.0000	0.571	0.0000
	32	0.448	0.746	0.0000	0.439	0.7354

NS5B	4	0.354	0.785	0.0000	0.756	0.0000
	8	0.361	0.739	0.0000	0.628	0.0000
	16	0.359	0.694	0.0000	0.485	0.0000
	32	0.365	0.687	0.0000	0.358	0.8151

I_ss_: index of substitution saturation; I_ss.c_: critical index of substitution saturation; *p*: *p* value for two-tailed tests.
